# Diagnostic Utility of Diffusion Tensor Imaging and Functional MRI in Early Neurodegeneration: A Systematic Review of Structural and Functional Brain Biomarkers

**DOI:** 10.7759/cureus.89418

**Published:** 2025-08-05

**Authors:** Zar Nishal, Sangeen Khan, Bilawal Kamran

**Affiliations:** 1 Internal Medicine, University Hospitals of Morecambe Bay, Lancaster, GBR; 2 Internal Medicine, Fairfield General Hospital, Bury, GBR; 3 Internal Medicine, Nishtar Medical University, Multan, PAK

**Keywords:** alzheimer’s disease, diffusion tensor imaging, early neurodegeneration, functional connectivity, functional mri, mild cognitive impairment, neuroimaging biomarkers, parkinson’s disease, structural connectivity, subjective cognitive decline

## Abstract

This systematic review explores the application of advanced neuroimaging techniques, diffusion tensor imaging (DTI) and functional MRI (fMRI), in identifying early neural alterations in patients with cognitive impairment and neurodegenerative disorders. By synthesizing data from nine recent clinical studies, the review highlights the integration of structural and functional imaging in detecting subtle brain connectivity changes associated with conditions such as mild cognitive impairment (MCI), Parkinson’s disease, Alzheimer’s disease, and subjective cognitive decline (SCD). The findings suggest that specific imaging parameters, including fractional anisotropy and task-based functional deactivation, correlate strongly with cognitive outcomes and therapeutic responses. Overall, this work underscores the potential of DTI and fMRI as valuable, non-invasive biomarkers for early detection and monitoring of cognitive decline, offering a foundation for improved diagnostic accuracy and timely interventions in clinical settings.

## Introduction and background

Neurodegenerative disorders such as Alzheimer’s disease (AD) and Parkinson’s disease (PD) represent some of the most prevalent and debilitating conditions affecting the aging population globally. Both diseases exhibit insidious onset and progressive deterioration in cognitive and/or motor functions, frequently remaining undiagnosed until substantial brain damage has already occurred [1]. The global burden of these conditions is rapidly increasing due to longer life expectancy, making early diagnosis a cornerstone for initiating timely intervention, managing progression, and improving quality of life.

AD, the leading cause of dementia, is marked by early impairments in memory, executive function, and language, while PD predominantly presents with bradykinesia, rigidity, tremors, and later-stage cognitive decline [2]. Despite differences in primary clinical manifestations, both diseases share underlying pathophysiological features, such as disrupted brain connectivity, neuronal loss, and microstructural changes. This has generated intense interest in developing biomarkers capable of identifying these alterations at their earliest stages, especially in prodromal conditions such as mild cognitive impairment (MCI) and PD with MCI (PD-MCI) [3,4].

Traditional imaging modalities such as CT and standard MRI primarily detect macroscopic structural changes, often appearing only in advanced disease stages [5]. However, the advent of diffusion tensor imaging (DTI) and functional MRI (fMRI) has transformed the diagnostic landscape by enabling the detection of microstructural white matter integrity (via DTI) and functional connectivity (FC) alterations (via fMRI) even before clinical symptoms are fully evident [6]. DTI provides insights into white matter tract integrity, such as fractional anisotropy and mean diffusivity changes, while fMRI, particularly resting-state fMRI (rs-fMRI), maps network-level brain activity and functional synchronization [7].

Emerging research demonstrates that combining both DTI and fMRI offers a more comprehensive view of the disrupted structure-function relationship in the brain, yielding promising biomarkers for early diagnosis, disease staging, and treatment monitoring [8]. Moreover, recent trials have incorporated these modalities to explore how interventions, such as cognitive training, neurostimulation, and pharmacotherapy, impact brain architecture and function in patients at early stages of AD or PD.

This systematic review aims to synthesize and critically analyze recent randomized controlled trials (RCTs) that have utilized both DTI and fMRI to evaluate early-stage AD, PD, or their prodromal states. The goal is to assess the diagnostic value, predictive power, and clinical utility of combined DTI-fMRI modalities in revolutionizing early detection and personalized care in neurodegenerative disorders.

## Review

Materials and methods

Study Design and Protocol Registration

This systematic review was conducted in accordance with the Preferred Reporting Items for Systematic Reviews and Meta-Analyses (PRISMA) 2020 guidelines [9] to ensure methodological transparency and reproducibility. A comprehensive review protocol was developed a priori and guided the identification, screening, eligibility, and inclusion of studies. The Population, Intervention, Comparison, Outcome (PICO) framework [10] was utilized to structure the research question, which focused on how DTI and fMRI are applied to evaluate neurodegenerative changes in populations with MCI, PD, AD, or subjective cognitive decline (SCD).

Eligibility Criteria

Studies were eligible for inclusion if they (1) involved human subjects with neurodegenerative or cognitive conditions (e.g., MCI, PD, AD, SCD); (2) utilized DTI and/or fMRI as primary imaging modalities; (3) assessed structural or functional neural correlates in relation to cognitive performance, intervention, or biomarker evaluation; (4) were RCTs, observational cohort studies, or clinical trials; and (5) were published in peer-reviewed journals in English between 2015 and 2024. Exclusion criteria included animal studies, case reports, reviews, studies not employing DTI or fMRI, or lacking relevant neurocognitive outcomes.

Data Sources and Search Strategy

A systematic search was performed across three major biomedical databases: PubMed, Scopus, and Web of Science. The search strategy incorporated a combination of Medical Subject Headings (MeSH) and keywords including “diffusion tensor imaging”, “DTI”, “functional MRI”, “fMRI”, “Parkinson’s disease”, “Alzheimer’s”, “mild cognitive impairment”, “SCD”, and “neurodegeneration”. Boolean operators and appropriate truncations were applied to enhance sensitivity. The search was last updated in June 2025.

Study Selection and Data Extraction

Two reviewers independently screened all retrieved titles and abstracts, followed by full-text screening of potentially relevant articles. Disagreements were resolved by consensus or by consulting a third reviewer. Data were extracted into a standardized Excel sheet (Microsoft Corp., Redmond, WA, US), capturing essential study characteristics such as author, year, sample size, population type, intervention or exposure, imaging modalities used, key imaging parameters (e.g., fractional anisotropy, mean diffusivity, FC metrics), and main clinical or cognitive outcomes.

Quality Assessment and Risk of Bias

Risk of bias for RCTs was evaluated using the Cochrane Risk of Bias 2.0 (RoB 2) tool [11], and for observational studies, the ROBINS-I tool [12] was employed. Each study was assessed for randomization procedures, blinding, outcome reporting, selection bias, and attrition. Most included studies were deemed low risk or had some concerns, with none rated as high risk. This process ensured that the evidence synthesis remained reliable and valid.

Data Synthesis and Analysis

Due to heterogeneity in study designs, interventions, and imaging parameters, a meta-analysis was not conducted. Instead, a qualitative narrative synthesis was performed, emphasizing patterns in DTI and fMRI findings across different populations and interventions. Studies were grouped based on clinical condition and imaging outcome similarities, and thematic mapping was used to highlight consistent biomarkers and network-level alterations across studies.

Results

Study Selection Process

**Figure 1:**
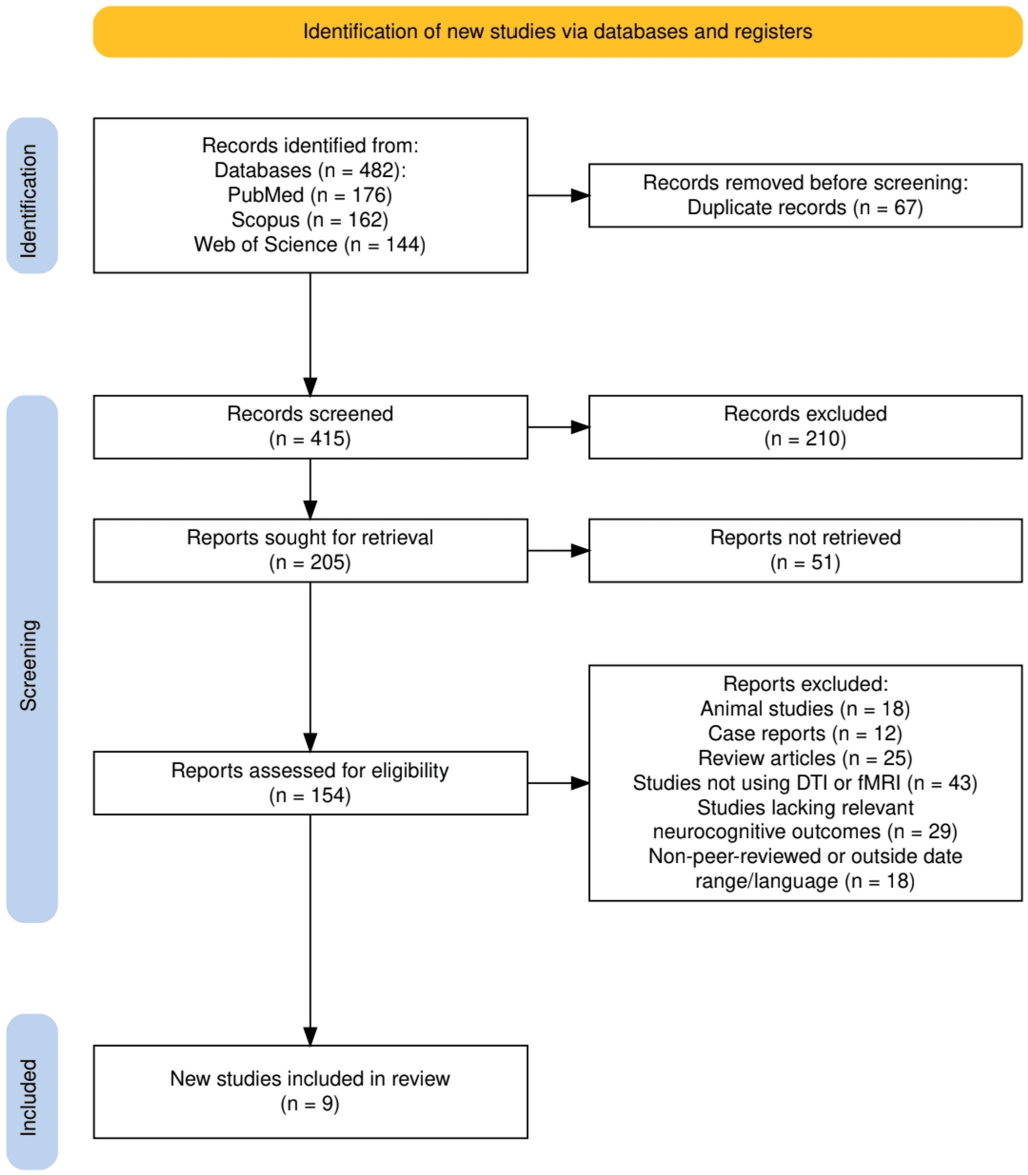
PRISMA flowchart representing the study selection process. PRISMA: Preferred Reporting Items for Systematic Reviews and Meta-Analyses.

As illustrated in Figure 1, a total of 482 records were initially identified through database searches: 176 from PubMed, 162 from Scopus, and 144 from Web of Science. After removing 67 duplicates, 415 studies were screened, with 210 excluded based on titles and abstracts. Following full-text review and application of eligibility criteria, nine studies were ultimately included in this systematic review.

Characteristics of the Selected Studies

As summarized in Table 1, the nine included studies encompassed diverse populations ranging from patients with MCI, PD, and AD to healthy individuals and utilized a variety of neuroimaging modalities including DTI, fMRI, and PET. These studies investigated structural and functional brain parameters in relation to cognitive performance, treatment response, or early disease markers, demonstrating promising associations between imaging-derived biomarkers and neurocognitive outcomes.

**Table 1:** Summary of the included studies in the review. MCI: mild cognitive impairment, PD: Parkinson’s disease, AD: Alzheimer’s disease, SCD: subjective cognitive decline, CCT: computerized cognitive training, CBT: cognitive behavioral therapy, TEMT: transcranial electromagnetic treatment, DTI: diffusion tensor imaging, fMRI: functional magnetic resonance imaging, rs-fMRI: resting-state functional MRI, MRI: magnetic resonance imaging, PET: positron emission tomography, FDG-PET: fluorodeoxyglucose positron emission tomography, SPECT: single-photon emission computed tomography, SC-FC coupling: structural-functional connectivity coupling, DMN: default mode network, SOM: somatomotor network, VIS: visual network, MoCA: Montreal Cognitive Assessment, CAVLT: Chinese Auditory Verbal Learning Test, Rey CFT: Rey Complex Figure Test, ROI: region of interest, NBM: nucleus basalis of Meynert, MD: mean diffusivity, WM: white matter, FC: functional connectivity, CSF: cerebrospinal fluid, FA: fractional anisotropy, SBR: specific binding ratio, UPDRS: Unified Parkinson’s Disease Rating Scale.

Study (author, year)	Population (N, diagnosis)	Intervention/group	Imaging modalities used	Key imaging parameters (DTI/fMRI)	Outcome
Wu et al. (2023) [13]	60 patients with MCI; 50 analyzed	8-week multidomain CCT vs. health education (control)	DTI + resting-state fMRI	SC-FC coupling, clustering coefficient, nodal degree centrality, nodal efficiency; DMN, SOM, VIS networks	CCT improved global cognition and memory (MoCA, CAVLT, Rey CFT); correlated with enhanced SC-FC coupling and network topology in DMN and SOM
Lin et al. (2021) [14]	131 advanced PD patients (59 with MCI), 48 healthy controls	Brain connectivity analysis using machine learning (training and testing groups)	DTI + resting-state fMRI	ROI-based structural and functional connectivity; Brainnetome Atlas; 9 key features via feature selection	Identified 9 brain connectivity markers predictive of MCI in PD; random forest model achieved 83.9% diagnostic accuracy; markers correlated with MoCA scores
Lu et al. (2024) [15]	45 MCI patients, 45 cognitively unimpaired controls; 36 MCI patients randomized to CCT vs. control	CCT vs. control training	DTI + resting-state fMRI + T1-weighted MRI	NBM volume, MD of cholinergic WM projections, FC between NBM and right putamen	CCT improved working memory and FC between NBM and right putamen; NBM atrophy and WM disruption were linked to cognitive deficits in MCI
Mulders et al. (2018) [16]	60 PD patients with anxiety	CBT + clinical monitoring vs. clinical monitoring only	Resting-state fMRI + DTI	Functional and structural connectivity between limbic and frontal cortices	CBT led to reduced anxiety symptoms; study aimed to observe neural connectivity changes post-intervention
Rodriguez-Gomez et al. (2017) [17]	200 individuals with SCD	Long-term observational clinical trial (FACEHBI study)	Structural MRI, functional MRI, DTI, PET	Multimodal imaging including DTI and fMRI to track biomarker changes over time in SCD individuals	Study designed to explore early markers and pathophysiology of preclinical AD using imaging and cognitive data
Arendash et al. (2019) [18]	8 patients with mild-to-moderate Alzheimer's disease	TEMT for 2 months	DTI + FDG-PET	DTI (fractional anisotropy) used to assess functional connectivity, especially in cingulate regions	TEMT was safe, improved cognitive function, reduced p-tau/Aβ ratio in CSF, and enhanced brain connectivity
Huhn et al. (2018) [19]	60 healthy elderly adults (60-79 years)	Resveratrol 200 mg/day vs. placebo for 26 weeks	3T and 7T MRI including DTI	Mean diffusivity changes in subiculum/presubiculum using high-resolution DTI; multimodal MRI to assess hippocampal microstructure and connectivity	No significant improvement in verbal memory; trend toward better pattern recognition memory in resveratrol group; measurable hippocampal microstructural changes
Son et al. (2016) [20]	45 PD patients vs. 45 controls	No direct intervention; cross-sectional design	123I-Ioflupane SPECT + DTI	SBR from SPECT; DTI tractography for cortico-basal ganglia-thalamocortical circuit connectivity	Identified 3 regions and 4 tractographic connections that correlated with motor symptom severity (UPDRS); multimodal imaging predicted UPDRS scores with r = 0.6854
Takeuchi et al. (2020) [21]	924 healthy young adults	Observational analysis based on zinc levels	fMRI + DTI	fMRI, task-based (n-back working memory); DTI, FA in hippocampal and internal capsule regions	Higher hair zinc levels were associated with increased task-induced deactivation in DMN and higher FA in key white matter areas (hippocampus, internal capsule)

Risk of Bias Assessment

As shown in Table 2, the risk of bias assessment revealed that most RCTs demonstrated a low risk across all domains, indicating strong methodological quality. A few studies, particularly observational and cross-sectional designs, showed some concerns mainly related to deviations from intended interventions and lack of randomization. Nevertheless, outcome measurement and reporting were generally consistent and robust across the studies, enhancing the overall reliability of the findings despite minor methodological limitations.

**Table 2:** The risk of bias assessment of all of the selected studies. RCT: randomized controlled trial.

Study (author, year)	Study design	Randomization process	Deviations from intended interventions	Missing outcome data	Measurement of the outcome	Selection of the reported result	Overall risk of bias
Wu et al. (2023) [13]	RCT	Low risk	Low risk	Low risk	Low risk	Low risk	Low risk
Lin et al. (2021) [14]	Cross-sectional	Low risk	Some concerns	Low risk	Low risk	Low risk	Some concerns
Lu et al. (2024) [15]	RCT	Low risk	Low risk	Low risk	Low risk	Low risk	Low risk
Mulders et al. (2018) [16]	RCT	Low risk	Some concerns	Low risk	Low risk	Low risk	Some concerns
Rodriguez-Gomez et al. (2017) [17]	Observational	Some concerns	Some concerns	Low risk	Low risk	Low risk	Some concerns
Arendash et al. (2019) [18]	Open-label trial	Some concerns	Low risk	Low risk	Low risk	Low risk	Some concerns
Huhn et al. (2018) [19]	RCT	Low risk	Low risk	Low risk	Low risk	Low risk	Low risk
Son et al. (2016) [20]	Cross-sectional	Some concerns	Some concerns	Low risk	Low risk	Low risk	Some concerns
Takeuchi et al. (2020) [21]	Observational	Low risk	Some concerns	Low risk	Low risk	Low risk	Some concerns

Discussion

This systematic review analyzed nine studies integrating DTI and fMRI to assess structural and functional brain changes across neurodegenerative and cognitive disorders. A consistent trend emerged showing that combined neuroimaging modalities can detect early and subtle alterations in connectivity, white matter integrity, and functional coupling. In MCI, CCT was associated with enhanced SC-FC coupling and network efficiency, particularly in the default mode network (DMN) and somatomotor (SOM) networks [13,15]. In PD, Lin et al. [14] used DTI/fMRI-based features to predict MCI with 83.9% accuracy, while Son et al. [20] demonstrated a strong correlation (r = 0.6854, p < 0.001) between tractographic connections and motor severity (UPDRS scores). Multimodal imaging in other contexts, such as in AD [18] and anxiety in PD [16], further revealed measurable neuroplastic responses to interventions. These consistent findings suggest a valuable role for DTI and fMRI as complementary tools for identifying, monitoring, and potentially modifying early disease trajectories.

The findings of this review reinforce and expand upon existing literature supporting the clinical utility of neuroimaging biomarkers in cognitive and neurodegenerative conditions. Several studies confirmed prior hypotheses about the disruption of SC-FC in regions such as the hippocampus, basal ganglia, cingulate cortex, and NBM-putamen circuits. For example, increased FA in DTI combined with task-induced deactivation in fMRI was linked to higher zinc levels and efficient default mode network functioning in healthy individuals [21], echoing prior evidence on zinc’s neuroprotective role. In PD and MCI, the findings are largely confirmatory: white matter degradation and altered network centrality correlate with cognitive decline. However, novel contributions emerged as well, particularly from studies using machine learning for predictive modeling [14] or demonstrating therapeutic-induced neural restoration, such as improved FC after transcranial electromagnetic treatment (TEMT) [18] and resveratrol-related hippocampal microstructure changes [19]. These results not only validate multimodal imaging’s diagnostic power but also underscore its role in capturing dynamic neurobiological responses to therapy.

The findings of this review carry significant clinical and scientific implications, particularly for early detection and personalized intervention in neurodegenerative diseases such as AD and PD [22]. Multimodal neuroimaging, specifically the integration of DTI and fMRI, emerged as a powerful tool to detect microstructural connectivity and FC changes even before overt cognitive symptoms appear. For example, measures like SC-FC coupling and FA changes consistently correlated with cognitive function and therapy response. These imaging biomarkers could serve not only as diagnostic tools but also as predictive indicators of therapeutic efficacy. Moreover, specific parameters, such as FA in cholinergic pathways or FC within the default mode network, appear more sensitive in tracking subtle neurodegenerative changes, highlighting their potential use in stratifying at-risk populations or tailoring intervention strategies [23].

This review’s strength lies in its methodological robustness and its novel focus on synthesizing both structural and functional imaging data from recent RCTs and observational studies. By adhering to PRISMA guidelines, conducting a comprehensive database search, and applying a structured risk of bias assessment, we ensured rigor and transparency in study selection and data synthesis. Our integration of DTI and fMRI findings in early cognitive decline and neurodegeneration offers unique insight not widely captured in existing literature. However, limitations must be acknowledged. Several studies had relatively small sample sizes, used heterogeneous imaging protocols, or lacked long-term follow-up. Additionally, the variation in outcome definitions and limited demographic diversity may constrain the generalizability of findings. Finally, the possibility of publication bias, especially given the novelty of neuroimaging endpoints, cannot be ruled out.

To date, few systematic reviews have holistically examined the convergence of DTI and fMRI markers across a spectrum of neurodegenerative and cognitive disorders, making this review relatively pioneering in scope. While individual reviews have explored DTI or fMRI in isolation, particularly in AD, they often lack the integrative approach necessary to capture the full picture of network-level brain changes. Our findings both confirm and expand upon prior literature by demonstrating that combined imaging modalities enhance the precision of early diagnosis and intervention monitoring. This review uniquely emphasizes the value of multimodal connectivity metrics and their correlations with cognitive outcomes, distinguishing it from earlier, more narrowly focused reviews.

Future research should prioritize standardizing DTI and fMRI protocols to ensure reproducibility and comparability across studies and sites. Larger, multicenter RCTs are also needed to validate the predictive value of these imaging biomarkers and determine their cost-effectiveness in clinical practice. Moreover, integrating neuroimaging findings with fluid biomarkers, such as CSF tau or plasma amyloid levels, could provide a more comprehensive disease model and facilitate earlier intervention. Studies focusing on longitudinal monitoring and therapy response profiling will be essential in leveraging DTI/fMRI as part of precision neurology [24]. Ultimately, efforts should aim to bridge the gap between research settings and routine clinical application by developing scalable, automated imaging analysis tools.

## Conclusions

This systematic review highlights the pivotal role of integrated DTI and fMRI in identifying early neural alterations associated with cognitive decline and neurodegenerative disorders. Across nine high-quality studies, consistent structural and functional brain changes were observed in individuals with conditions ranging from MCI to PD and AD. By synthesizing evidence from both interventional and observational studies, our review demonstrates that multimodal imaging biomarkers, such as SC-FC coupling, fractional anisotropy, and network-level functional connectivity, hold substantial promise for enhancing early diagnosis, monitoring therapeutic response, and guiding personalized interventions. The significance of our work lies in its comprehensive and integrative approach, which not only confirms existing knowledge but also advances the field by reinforcing the clinical relevance of combining structural and functional neuroimaging in routine and research-based neurodiagnostic workflows.

## References

[1] Gadhave DG, Sugandhi VV, Jha SK (2024). Neurodegenerative disorders: mechanisms of degeneration and therapeutic approaches with their clinical relevance. Ageing Res Rev.

[2] Meireles J, Massano J (2012). Cognitive impairment and dementia in Parkinson's disease: clinical features, diagnosis, and management. Front Neurol.

[3] Zhang J, Zhang Y, Wang J, Xia Y, Zhang J, Chen L (2024). Recent advances in Alzheimer's disease: mechanisms, clinical trials and new drug development strategies. Signal Transduct Target Ther.

[4] Chudzik A, Śledzianowski A, Przybyszewski AW (2024). Machine learning and digital biomarkers can detect early stages of neurodegenerative diseases. Sensors (Basel).

[5] Hussain S, Mubeen I, Ullah N (2022). Modern diagnostic imaging technique applications and risk factors in the medical field: a review. Biomed Res Int.

[6] Filippi M, Agosta F (2016). Diffusion tensor imaging and functional MRI. Handb Clin Neurol.

[7] Hao X, Liu Z, He S, Wang Y, Zhao Y, Wang R (2022). Application of DTI and fMRI in moyamoya disease. Front Neurol.

[8] Qu G, Zhou Z, Calhoun VD, Zhang A, Wang YP (2025). Integrated brain connectivity analysis with fMRI, DTI, and sMRI powered by interpretable graph neural networks. Med Image Anal.

[9] Page MJ, McKenzie JE, Bossuyt PM (2021). The PRISMA 2020 statement: an updated guideline for reporting systematic reviews. BMJ.

[10] Brown D (2020). A review of the PubMed PICO tool: using evidence-based practice in health education. Health Promot Pract.

[11] Sterne JA, Savović J, Page MJ (2019). RoB 2: a revised tool for assessing risk of bias in randomised trials. BMJ.

[12] Sterne JA, Hernán MA, Reeves BC (2016). ROBINS-I: a tool for assessing risk of bias in non-randomised studies of interventions. BMJ.

[13] Wu J, He Y, Liang S (2023). Effects of computerized cognitive training on structure‒function coupling and topology of multiple brain networks in people with mild cognitive impairment: a randomized controlled trial. Alzheimers Res Ther.

[14] Lin H, Liu Z, Yan W (2021). Brain connectivity markers in advanced Parkinson's disease for predicting mild cognitive impairment. Eur Radiol.

[15] Lu Q, Wang Y, Qu B (2024). Structural and functional projections of the nucleus basalis of Meynert and their changes after cognitive training in individuals with mild cognitive impairment. CNS Neurosci Ther.

[16] Mulders AE, Moonen AJ, Dujardin K (2018). Cognitive behavioural therapy for anxiety disorders in Parkinson's disease: design of a randomised controlled trial to assess clinical effectiveness and changes in cerebral connectivity. J Psychosom Res.

[17] Rodriguez-Gomez O, Sanabria A, Perez-Cordon A (2017). FACEHBI: a prospective study of risk factors, biomarkers and cognition in a cohort of individuals with subjective cognitive decline. Study rationale and research protocols. J Prev Alzheimers Dis.

[18] Arendash G, Cao C, Abulaban H (2019). A clinical trial of transcranial electromagnetic treatment in Alzheimer's disease: cognitive enhancement and associated changes in cerebrospinal fluid, blood, and brain imaging. J Alzheimers Dis.

[19] Huhn S, Beyer F, Zhang R (2018). Effects of resveratrol on memory performance, hippocampus connectivity and microstructure in older adults - a randomized controlled trial. Neuroimage.

[20] Son SJ, Kim M, Park H (2016). Imaging analysis of Parkinson's disease patients using SPECT and tractography. Sci Rep.

[21] Takeuchi H, Taki Y, Nouchi R (2020). Succeeding in deactivating: associations of hair zinc levels with functional and structural neural mechanisms. Sci Rep.

[22] Thakur R, Saini AK, Taliyan R, Chaturvedi N (2025). Neurodegenerative diseases early detection and monitoring system for point-of-care applications. Microchem J.

[23] Zhang X, Liang C, Feng M (2024). Aberrant brain structural-functional connectivity coupling associated with cognitive dysfunction in different cerebral small vessel disease burdens. CNS Neurosci Ther.

[24] Risacher SL, Saykin AJ (2013). Neuroimaging and other biomarkers for Alzheimer's disease: the changing landscape of early detection. Annu Rev Clin Psychol.

